# Inverse association between low-density lipoprotein cholesterol and bone mineral density in young- and middle-aged people: The NHANES 2011–2018

**DOI:** 10.3389/fmed.2022.929709

**Published:** 2022-08-10

**Authors:** Fangjun Xiao, Peng Peng, Shihua Gao, Tianye Lin, Weihua Fang, Wei He

**Affiliations:** ^1^Guangzhou University of Chinese Medicine, Guangzhou, China; ^2^Guangdong Research Institute for Orthopedics and Traumatology of Chinese Medicine, Guangzhou, China; ^3^Joint Center, The Third Affiliated Hospital of Guangzhou University of Chinese Medicine, Guangzhou, China

**Keywords:** low-density lipoprotein cholesterol, bone mineral density, National Health and Nutrition Examination Survey (NHANES), young- and middle-aged people, osteoporosis

## Abstract

**Objectives:**

Low-density lipoprotein cholesterol (LDL-C) plays an essential part in bone metabolism. However, the correlation between LDL-C levels and bone mineral density (BMD) is still controversial. This study aimed to explore the relationship between LDL-C levels and lumbar BMD in young- and middle-aged people.

**Methods:**

We conducted a cross-sectional study comprising 4,441 participants aged 20–59 from the National Health and Nutrition Examination Survey (NHANES). LDL-C levels and lumbar BMD were used as independent and dependent variables, respectively. We evaluated the correlation between LDL-C levels and lumbar BMD through a weighted multivariate linear regression model. We performed a subgroup analysis of the relationship between LDL-C levels and lumbar BMD based on age, gender, and body mass index (BMI).

**Results:**

After adjusting for confounding factors, LDL-C levels were negatively correlated with lumbar BMD. In subgroup analyses stratified by gender, this negative association was statistically significant in men and women. In the subgroup analysis stratified by age, a negative connection existed in people aged 30–49 years. In the subgroup analysis divided by BMI, there was an inverse correlation in overweight people (25 ≤ BMI < 30).

**Conclusions:**

Our research observed an inverse association between LDL-C levels and lumbar BMD in young- and middle-aged people, especially in people aged 30–49 years and who are overweight. Close monitoring of BMD and early intervention may be required for these people.

## Introduction

Osteoporosis is a common bone metabolic disease characterized by low bone mass and disruption of bone architecture. According to a study by International Osteoporosis Foundation, one-third of women and one-fifth of men over the age of 50 suffer from osteoporosis and are at risk of osteoporotic fractures (1). Even more concerning is the fact that osteoporosis is often undiagnosed until patients experience fragility fractures (2). Currently, the reduction of bone mineral density (BMD) is a vital diagnosis standard of osteoporosis (3, 4). Most osteoporotic fractures are entirely preventable if loss of bone mass can be detected and prevented in the early stages.

Osteoporosis was associated with many factors, such as genetic and environmental factors (5). They can individually or synergistically lead to a decrease in bone mass and promote the development of osteoporosis progression (6). Assessing the risk factor of osteoporosis can effectively reduce the incidence of fragility fractures. In recent studies, multiple lines of evidence suggested that lipid metabolism simultaneously involves the progression of osteoporosis and cardiovascular disease (7). Meanwhile, dyslipidemia might effectively predict osteoporosis (8, 9). Low-density lipoprotein cholesterol (LDL-C), a recognized hazard factor for cardiovascular disease, participates in the formation of atherosclerotic plaques (10). However, there is no definite answer to the relationship between LDL-C levels and osteoporosis.

National Health and Nutrition Examination Survey (NHANES) has a long history, beginning in the early 1960s as the National Health Examination Survey that continuously collected information on the health and nutrition of the US household population. NHANES provides data to describe dietary intakes and prevalence estimates for risk factors and selected diet-related and other diseases in the US population. These data are critical for exploring the emerging public health needs of the nation (11). Our study aimed to investigate the association of LDL-C levels with lumbar BMD based on the existing NHANES dataset to provide clinicians with statistical data for early intervention in patients with a high probability of developing osteoporosis.

## Methods

### Data collection and study population

**Figure 1:**
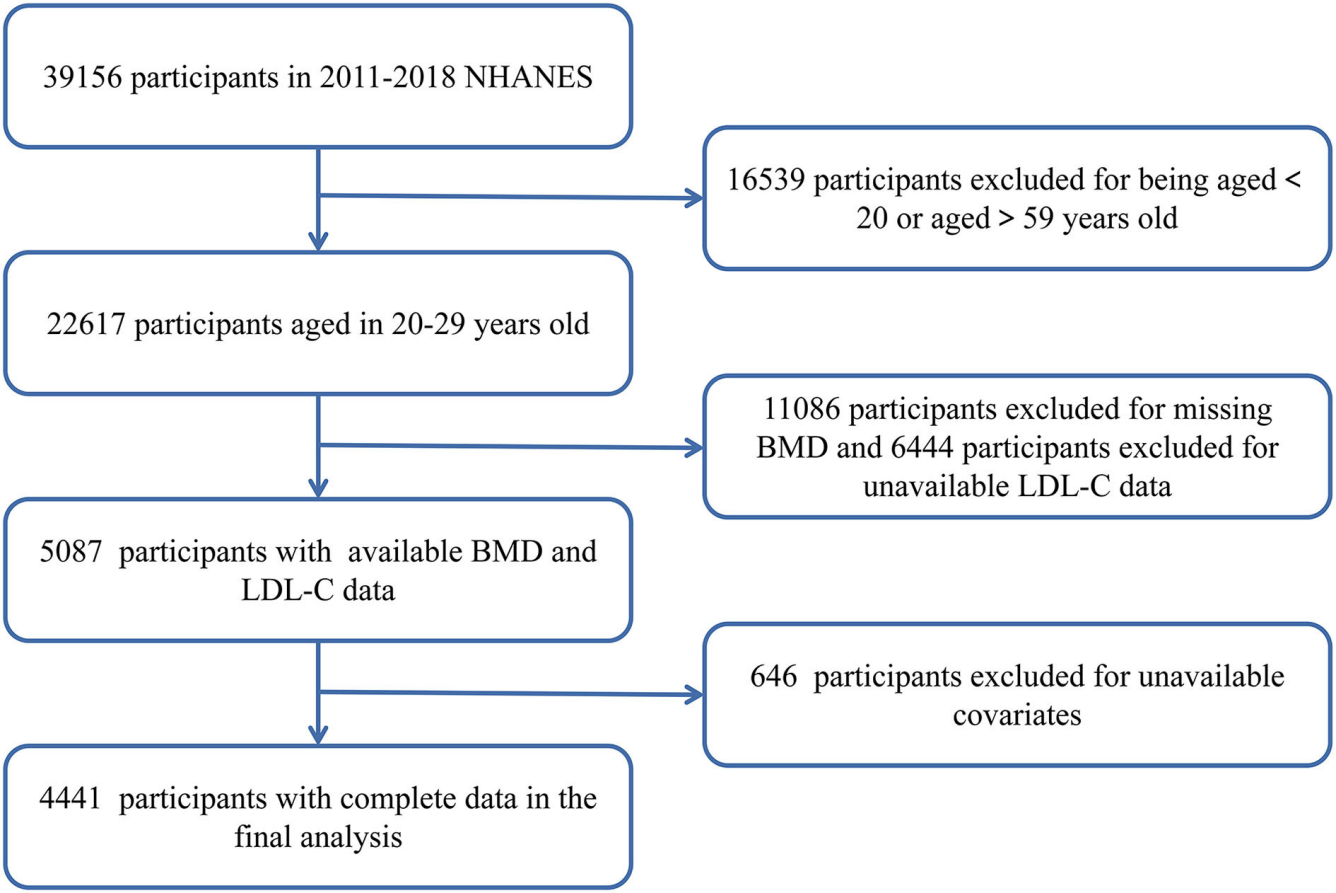
Flowchart of sample selection.

National Health and Nutrition Examination Survey was approved by NCHS Institutional Review Board, and informed consent from all participants was obtained (10). In our study, we collected data in four consecutive NHANES cycles (2011–2012, 2013–2014, 2015–2016, and 2017–2018). A total of 39,156 participants were included. After screening of participants aged 20–59 years (*n* = 22,618) and exclusion of participants with unavailable BMD data (*n* = 11,086), unavailable LDL-C data (*n* = 6,444), unavailable 24-h diet recall data (*n* = 247), and unavailable other covariate data (*n* = 399), 4,441 participants were finally included in the analysis (Figure 1).

### Variables

In this study, the independent variable was LDL-C. Plasma cholesterol levels were measured on participants who were examined in the morning. Serum LDL-C levels were calculated from directly measured values of total cholesterol, triglycerides, and high-density lipoprotein cholesterol (HDL-C) in accordance with Friedewald's calculation formula: [LDL-C] = [total cholesterol] – [HDL-C] – [triglycerides/5] (12). The dependent variable was the lumbar BMD, which was measured by the dual-energy X-ray bone densitometer. The analysis included the following covariates: age, gender, race, education level, marital status, history of hypertension, history of diabetes, smoking, body mass index (BMI), poverty-to-income ratio, total calcium intake, and total protein intake. The examination parts of clinical, physiological, and laboratory evaluations were carried out by well-trained medical experts. The detailed collection procedures and measurement methods for each variable are publicly available at www.cdc.gov/nchs/nhanes.

### Statistical analysis

All assessments were calculated by accounting for NHANES sampling weights. The characteristics of the study population involved in the final analysis were described by the mean ± standard deviation (SD; continuity variable) or percentages (categorical variable). Weighted multivariate linear regression models were used to evaluate the linear relationship between LDL-C levels and lumbar BMD. We standardized LDL-C levels with a mean of 0 and an SD of 1 and constructed three models (13). In the unadjusted model, no covariates were adjusted. The minimally adjusted model was adjusted for age, gender, and race. The fully adjusted model were additionally adjusted for education level, marital status, income poverty ratio, BMI, smoking behavior, hypertension, diabetes, total calcium intake, and total protein intake based on the minimally adjusted model. Stratified analysis was conducted according to gender, age, and BMI to determine the potential subgroup with a significant correlation between LDL-C levels and BMD. The *p*-values <0.05 were considered statistically significant. Data analysis was performed with the statistical software packages R (version: 4.0.3, http://www.R-project.org) and EmpowerStats (version: 2.0, http://www.empowerstats.com).

## Result

### Characteristics of the study population

Our study involved 4,441 participants aged 20–59 years. Table 1 describes weighted sociodemographic and medical characteristics based on the quartiles of LDL-C levels. There was no statistically significant association between the distribution of race and total protein intake. Participants in the highest quartile of LDL-C levels were more likely to be men (54.46%), with high school education or above (62.78%), to be married (58.45%), to be overweight (BMI 29.89 ± 6.28), and have a higher family income to poverty ratio (3.02 ± 1.65). Participants who did not have hypertension (73.41%), did not have diabetes (92.26%), and were non-smokers (55.61%) had higher LDL-C levels. Meanwhile, lower total calcium intake (83.91 ± 33.87) and lower lumbar BMD (1.01 ± 0.14) correlated with higher LDL-C levels.

**Table 1:** Weighted characteristics of the study population based on low-density lipoprotein cholesterol (LDL-C) quartiles.

**LDL cholesterol (mg/dL)**	**Total**	**Q1**	**Q2**	**Q3**	**Q4**	***P*-value**
Age (years)	39.72 ± 11.80	35.27 ± 11.95	38.61 ± 11.89	41.23 ± 11.57	43.32 ± 10.26	<0.0001
Gender (%)						0.0049
Men	51.97	47.69	51.15	54.19	54.46	
Women	48.03	52.31	48.85	45.81	45.54	
Race (%)						0.2000
Mexican American	9.45	9.10	9.62	10.95	8.16	
Other Hispanic	6.72	5.71	6.29	7.70	7.13	
White	63.84	62.83	64.09	62.96	65.34	
Black	10.91	12.99	10.48	9.66	10.68	
Other Race	9.07	9.36	9.52	8.73	8.69	
Education level (%)						0.0444
Under high school	12.86	11.08	13.72	13.28	13.17	
High school or equivalent	21.56	21.52	18.96	21.69	24.05	
Above high school	65.59	67.40	67.32	65.04	62.78	
Marital status (%)						<0.0001
Married	51.97	44.32	48.50	55.92	58.45	
Widowed	1.28	1.31	0.66	0.87	2.25	
Divorced	9.70	7.10	8.98	10.33	12.12	
Separated	2.41	1.88	2.30	3.05	2.38	
Never married	23.90	32.37	28.17	20.36	15.52	
Living with partner	10.74	13.01	11.39	9.47	9.29	
Hypertension (%)						0.0176
YES	24.19	21.32	25.44	23.00	26.59	
NO	75.81	78.68	74.56	77.00	73.41	
Diabetes (%)						<0.0001
YES	6.09	9.05	5.05	5.57	5.02	
NO	92.14	89.11	93.94	92.95	92.26	
Borderline	1.77	1.84	1.01	1.48	2.72	
Smoked at least 100 cigarettes in life (%)						0.0211
YES	41.59	37.85	41.83	41.87	44.39	
NO	58.41	62.15	58.17	58.13	55.61	
BMI (kg/m^2^)	29.05 ± 6.93	27.88 ± 7.35	28.43 ± 6.78	29.89 ± 7.11	29.89 ± 6.28	<0.0001
Ratio of family income to Poverty	2.90 ± 1.66	2.69 ± 1.66	2.92 ± 1.68	2.96 ± 1.64	3.02 ± 1.65	<0.0001
Total calcium intake (mg/d)	1081.15 ± 576.91	1113.16 ± 655.64	1094.10 ± 581.30	1090.75 ± 546.72	1030.90 ± 520.04	0.0049
Total protein intake (mg/d)	85.24 ± 36.67	85.73 ± 39.10	85.39 ± 37.08	86.02 ± 36.71	83.91 ± 33.87	0.5254
Lumbar BMD (g/cm^2^)	1.03 ± 0.14	1.05 ± 0.15	1.04 ± 0.15	1.03 ± 0.14	1.01 ± 0.14	<0.0001

Mean ± SD for continuous variables: p-value was calculated by the weighted linear regression model. % for categorical variables: p-value was calculated by weighted chi-square test.

### Association between LDL-C and lumbar BMD

The results of multivariate regression analysis showed that LDL-C levels were negatively correlated with lumbar BMD (*β* = −0.014, 95% CI: −0.018, −0.010) in the unadjusted model. After adjusting for confounding factors, this negative correlation still existed in the minimally adjusted model (*β* = −0.012, 95% CI: −0.016, −0.007) and the fully adjusted model (*β* = −0.010, 95% CI: −0.015, −0.006). Meanwhile, there was a linear correlation in the fully adjusted model (*p* for trend <0.05). The lumbar BMD of the highest quartile was 0.026 g/cm^2^ lower than that of the lowest quartile (Table 2).

**Table 2:** Association between low-density lipoprotein cholesterol (LDL-C; mg/dl) and lumbar body mass density (BMD; g/cms^2^) among young- and middle-aged people.

**Models**	**Non-adjusted model** **β (95% CI) *P*-value**	**Minimally-adjusted model** **β (95% CI) *P*-value**	**Fully-adjusted model** **β (95% CI) *P-*value**
LDL CHOLESTEROL(mg/dL)	−0.014 (−0.018, −0.010) ^***^	−0.012 (−0.016, −0.007) ^***^	−0.010 (−0.015, −0.006) ^***^
LDL CHOLESTEROL (quartile)			
Q1	Reference	Reference	Reference
Q2	−0.013 (−0.025, −0.001) ^*^	−0.008 (−0.020, 0.004)	−0.006 (−0.018, 0.006)
Q3	−0.026 (−0.038, −0.013) ^***^	−0.018 (−0.030, −0.005) ^**^	−0.016 (−0.028, −0.004) ^*^
Q4	−0.037 (−0.050, −0.025) ^***^	−0.030 (−0.042, −0.018) ^***^	−0.026 (−0.039, −0.014) ^***^
*P* for trend	<0.001	<0.001	<0.001

Non-adjusted model: no covariates were adjusted. Minimally adjusted model: age, gender, and race were adjusted. Fully adjusted model: age, gender, race, education level, marital status, income poverty ratio, BMI, smoking behavior, hypertension, diabetes, total calcium intake, and total protein intake were adjusted.

* p < 0.05,

** p < 0.01,

*** p < 0.001.

In subgroup analysis based on gender stratification, the negative correlation between LDL-C levels and lumbar BMD existed in men (*β* = −0.009, 95% CI: −0.015, −0.003) and women (*β* = −0.009, 95% CI: −0.015, −0.003) in the fully adjusted model (Table 3).

**Table 3:** Association between low-density lipoprotein cholesterol (LDL-C; mg/dl) and lumbar body mass density (BMD; g/cm^2^) stratified by gender.

**Stratified by gender**	**Non-adjusted model** **β (95% CI) *P*-value**	**Minimally-adjusted model** **β (95% CI) *P*-value**	**Fully-adjusted model** **β (95% CI) *P-*value**
Men	−0.013 (−0.020, −0.007) ^***^	−0.013 (−0.019, −0.006) ^***^	−0.009 (−0.015, −0.003) ^**^
Women	−0.015 (−0.021, −0.009) ^***^	−0.009 (−0.015, −0.003) ^**^	−0.009 (−0.015, −0.003) ^**^

Non-adjusted model: no covariates were adjusted. Minimally adjusted model: age and race were adjusted. Fully adjusted model: age, race, education level, marital status, income poverty ratio, BMI, smoking behavior, hypertension, diabetes, total calcium intake, and total protein intake were adjusted.

** p < 0.01,

*** p < 0.001.

The subgroup analysis based on age stratification showed that LDL-C levels were inversely associated with lumbar BMD in people aged 30–59 years in the non-adjusted and minimally adjusted models (*p* < 0.05). In the fully adjusted model, there was no significant correlation between LDL-C levels and lumbar BMD in people aged 50–59 years. In all models, LDL-C levels had no association with lumbar BMD in people aged 20–29 years (Table 4).

**Table 4:** Association between low-density lipoprotein cholesterol (LDL-C; mg/dl) and lumbar body mass density (BMD; g/cm^2^) stratified by age.

**Stratified by age** **(years)**	**Non-adjusted model** **β (95% CI) *P*-value**	**Minimally-adjusted model** **β (95% CI) *P*-value**	**Fully-adjusted model** **β (95% CI) *P-*value**
20–29	−0.005 (−0.014, 0.003)	−0.005 (−0.013, 0.004)	−0.004 (−0.013, 0.005)
30–39	−0.015 (−0.024, −0.006) ^**^	−0.011 (−0.020, −0.002) ^*^	−0.009 (−0.019, −0.000) ^*^
40–49	−0.018 (−0.027, −0.009) ^***^	−0.015 (−0.024, −0.007) ^***^	−0.014 (−0.023, −0.006) ^**^
50–59	−0.014 (−0.022, −0.006) ^***^	−0.011 (−0.019, −0.003) ^**^	−0.006 (−0.014, 0.002)

Non-adjusted model: no covariates were adjusted. Minimally adjusted model: gender and race were adjusted. Fully adjusted model: gender, race, education level, marital status, income poverty ratio, BMI, smoking behavior, hypertension, diabetes, total calcium intake, and total protein intake were adjusted.

* p < 0.05,

** p < 0.01,

*** p < 0.001.

Body mass index is a factor closely related to BMD and LDL-C levels (14). The subgroup analysis based on BMI stratification showed a negative correlation between LDL-C levels and lumbar BMD in the unadjusted model. Meanwhile, we found a strong correlation between LDL-C levels and lumbar BMD in people who are overweight (25 ≤ BMI < 30; *p* < 0.001). In the fully adjusted model, lumbar BMD decreased by 0.017 g/cm^2^ per 1 SD while LDL-C levels increased in people who are overweight (Table 5).

**Table 5:** Association between low-density lipoprotein cholesterol (LDL-C; mg/dl) and lumbar body mass density (BMD; g/cm^2^) stratified by body mass index (BMI).

**Stratified by BMI** **(kg/m^2^)**	**Non-adjusted model** **β (95% CI) *P-*value**	**Minimally-adjusted model** **β (95% CI) *P*-value**	**Fully-adjusted model** **β (95% CI) *P-*value**
Healthy Weight (BMI < 25)	−0.013 (−0.021, −0.006) ^***^	−0.007 (−0.015, 0.001)	−0.007 (−0.015, 0.001)
Overweight (25 ≤ BMI < 30)	−0.021 (−0.029, −0.014) ^***^	−0.019 (−0.027, −0.012) ^***^	−0.017 (−0.025, −0.010) ^***^
Obesity (BMI ≥ 30)	−0.011 (−0.019, −0.004) ^**^	−0.010 (−0.018, −0.003) ^**^	−0.005 (−0.012, 0.003)

Non-adjusted model: no covariates were adjusted. Minimally adjusted model: age, gender, and race were adjusted. Fully adjusted model: age, gender, race, education level, marital status, income poverty ratio, smoking behavior, hypertension, diabetes, total calcium intake, and total protein intake were adjusted.

** p < 0.01,

*** p < 0.001.

## Discussion

In an analysis of four merged datasets from 2011 to 2018 NHANES surveys, LDL-C levels were inversely correlated with lumbar BMD. People with higher levels of LDL-C levels were more likely to be 39.72 ± 11.80 years old, be of male gender, be married, be overweight, and have higher family income to poverty ratio. We performed subgroup analysis based on gender, age, and BMI. We found that this negative association was statistically significant in men and women. In the subgroup analysis stratified by age, a negative connection exists in people aged 30–49 years. In the subgroup analysis divided by BMI, there was an inverse correlation in people who are overweight.

Bones are active endocrine organs carrying several metabolic functions (15). Several studies detected the correlation between dyslipidemia and BMD (16, 17). However, the research results are debatable. Zhang et al. reported that, when LDL-C levels were <3.52 mmol/L values, LDL-C levels were negatively correlated with lumbar BMD in Chinese postmenopausal women (18). Other studies discovered a positive relationship between HDL-C and lumbar BMD and suggested that lipid metabolism plays a vital role in regulating BMD (19, 20). However, Makovey et al. observed that HDL-C levels were inversely associated with BMD in premenopausal women (21). Martín-Gonzalez et al. found that BMD was positively correlated with LDL-C levels in a cross-sectional study of 280 individuals with chronic alcohol consumption (22). Sivas et al. demonstrated that triglyceride was not associated with BMD in postmenopausal Turkish women (23). These counterintuitive observations supplement other conclusions drawn from several similar studies conducted on different populations. Nonetheless, most studies reported that LDL-C levels were inversely associated with BMD (13, 24, 25). Our research found that lower LDL-C levels may be associated with higher lumbar BMD.

Traditionally, osteoporosis has been regarded as a disease of postmenopausal women; nevertheless, osteoporosis in men is a frequent and severe condition (26). Our research found that LDL-C levels were negatively correlated with lumbar BMD in men and women. In the fully adjusted model, men and women share a consistent *β* value. Previous studies where oxidized LDL-C directly impede differentiation of osteoblasts suggested lipid profiles as a probable risk factor for osteoporosis (27, 28). In addition, evidence accumulated over the past years strongly suggests that estrogen also plays a critical role in regulating the male skeleton (29). Hence, whether LDL-C affects BMD by regulating estrogen deserves further study.

Aging is a known risk factor associated with deterioration of bone mass, leading to an increased risk of fragility fractures (30). Most cohort and cross-sectional studies focused on BMD in older adults or postmenopausal women (31, 32). However, it is increasingly clear that bone mass acquired during growth is also a vital determinant of fragility fracture resistance in the future (33). When we performed a subgroup analysis based on age, we found a statistically inverse association between LDL-C levels and lumbar BMD in people aged 30–49 years in the fully adjusted model. The correlation is also stronger with increasing age. Hence, our findings may provide insights for strategies to prevent osteopenia in this age group.

Obesity is another prevalent public health problem with strong clinical links to osteoporosis, and BMI is a simple way of measuring the degree of obesity (34). Some previous studies found a positive association between BMI and BMD. Lower BMI is associated with a greater risk of osteoporosis (35). However, recent epidemiological and clinical research has challenged this belief. Some studies reported a significant percentage of fragility fractures in women who are obese due to the body structure or mechanism of injury (36, 37). Our research found a statistically inverse association between LDL-C and lumbar BMD in overweight people but not in other subgroups in the fully adjusted model. However, the specific mechanism still needs further research.

The strength of this study is that our research examined the association between LDL-C levels and lumbar BMD by using large epidemiology data. Additionally, the inverse connection between LDL-C levels and lumbar BMD remained statistically significant after adjustment for potential confounders in multivariate linear regression models. However, it is important to acknowledge the limitations of our study. First, this study has taken American participants as the research object. It is uncertain whether the link between LDL-C levels and BMD is applicable to other countries or races due to genetic, environmental, and cultural differences. Second, the cross-sectional study design has made it impossible to determine the causal relationship between LDL-C levels and BMD. Therefore, further prospective clinical and basic experimental studies are necessary to determine the exact mechanism of the association between LDL-C levels and BMD.

## Conclusions

Our research observed a negative association between LDL-C levels and lumbar BMD in young- and middle-aged people, especially in people aged 30–49 years and who are overweight. Close monitoring of BMD and early intervention may be required for these people.

## Data availability statement

The datasets presented in this study can be found in online repositories. The names of the repository/repositories and accession number(s) can be found below: https://wwwn.cdc.gov/nchs/nhanes/.

## Ethics statement

The studies involving human participants were reviewed and approved by the board of the National Center for Health Ethics Review Board. The patients/participants provided their written informed consent to participate in this study.

## Author contributions

FX and PP: drafting of the manuscript. WF: data collection and analysis. TL and SG: review and editing. WH: study concept and design. All authors contributed to the article and approved the submitted version.

## Funding

This work was supported by the National Natural Science Foundation of China (Grant numbers 81873327, 82004392, and 81573996), the Double First-class Discipline Construction Project of Guangzhou University of Chinese Medicine (grant number Z2015002), the major project of “Double First-class” and High-level University Discipline Collaborative Innovation Team of Guangzhou University of Chinese Medicine (grant number 2021XK05), the cultivated project of “Double First-class” and High-level University Discipline Collaborative Innovation Team of Guangzhou University of Chinese Medicine (grant numbers 2021XK41 and 2021XK46), and the Foundation of Guangdong Educational Committee for Youth Scientists (grant number 2019KQNCX017).

## Conflict of interest

The authors declare that the research was conducted in the absence of any commercial or financial relationships that could be construed as a potential conflict of interest.

## Publisher's note

All claims expressed in this article are solely those of the authors and do not necessarily represent those of their affiliated organizations, or those of the publisher, the editors and the reviewers. Any product that may be evaluated in this article, or claim that may be made by its manufacturer, is not guaranteed or endorsed by the publisher.
